# A 4-gene signature predicts prognosis of uterine serous carcinoma

**DOI:** 10.1186/s12885-021-07834-4

**Published:** 2021-02-12

**Authors:** Hui Chen, Lingjun Li, Ping Qin, Hanzhen Xiong, Ruichao Chen, Minfen Zhang, Qingping Jiang

**Affiliations:** 1 Department of Pathology, School of Basic Medical Science, Guangzhou Medical University, Guangzhou, China; 2 Department of Pathology, Third Affiliated Hospital, Guangzhou Medical University, Guangzhou, China

**Keywords:** Uterine serous carcinoma, Biomarkers, Signature, Mortality, Recurrence

## Abstract

**Background:**

Uterine serous carcinoma (USC) is an aggressive type of endometrial cancer that accounts for up to 40% of endometrial cancer deaths, creating an urgent need for prognostic biomarkers.

**Methods:**

USC RNA-Seq data and corresponding patients’ clinical records were obtained from The Cancer Genome Atlas and Genotype-Tissue Expression datasets. Univariate cox, Lasso, and Multivariate cox regression analyses were conducted to forge a prognostic signature. Multivariable and univariable cox regression analysis and ROC curve evaluated the prediction efficiency both in the training and testing sets.

**Results:**

We uncovered 1385 genes dysregulated in 110 cases of USC tissue relative to 113 cases of normal uterine tissue. Functional enrichment analysis of these genes revealed the involvement of various cancer-related pathways in USC. A novel 4-gene signature (KRT23, CXCL1, SOX9 and ABCA10) of USC prognosis was finally forged by serial regression analyses. Overall patient survival (OS) and recurrence-free survival (RFS) were significantly lower in the high-risk group relative to the low-risk group in both the training and testing sets. The area under the ROC curve of the 4-gene signature was highest among clinicopathological features in predicting OS and RFS. The 4-gene signature was found to be an independent prognostic indicator in USC and was a superior predictor of OS in early stage of USC.

**Conclusions:**

Our findings highlight the potential of the 4-gene signature as a guide for personalized USC treatment.

**Supplementary Information:**

The online version contains supplementary material available at 10.1186/s12885-021-07834-4.

## Background

Endometrial cancer is the 2nd most common gynecologic malignancy worldwide [1]. In China it also ranks the 2nd most common female cancer of the genital tract [2]. Uterine serous carcinoma (USC/uterine serous papillary carcinoma) was first described by Hendrickson in 1982 [3]. It represents a type of endometrial cancer whose clinicopathological and molecular features deviate from those of endometrioid carcinoma (EEC). Unlike EEC, USC tends to develop in elderly women, with low body weight and arises in the background of atrophic endometrium [4]. Microscopically, USC typically forms complex papillary structure with almost high-grade polymorphic nuclei in contrast to glandular/cribriform pattern with mild to moderate atypical nuclei in EEC [5, 6]. 80–90% of USC tumors harbor TP53 mutation while retaining wildtype PTEN but losing ER/PR expression [5–8]. USC accounts for almost 10% of endometrial cancers but is disproportionately responsible for poor outcomes, contributing up to 40% cancer-related deaths from endometrial cancer [4]. The estimated 5-year disease-specific survival for USC is 18–27% compared with that of 80–90% for EEC. Compared in stage, USC has better 5-year disease-specific survival than grade 3 EEC both in early (stage I/II, 74% vs. 85%, *p* < 0.0001) and late stage (stage III/IV, 33% vs. 54%, *p* < 0.0001) [9]. USC is characteristically aggressive, readily invading lymph-vascular space and undergoing abdominal dissemination in the early and stages even in the absence of myometrium invasion [10–15]. A high proportion of USC cases present with extrauterine symptoms and adnexal, peritoneal or upper abdominal mass at diagnosis [16–18]. Therefore, the clinicopathological parameters that can predict the prognosis of EEC, such as tumor size, myometrial invasion, lymph-vascular space invasion and lymph node metastasis, are not reliable indicators of USC prognosis [4, 19, 20]. To the best of our knowledge, a robust system for predicting USC outcomes and recurrence is currently unavailable.

Advancements in molecular biological techniques and RNA-sequencing technology, have made it easier to identify genes that are associated with cancer initiation and progression [21]. Single or multiple gene signatures exhibiting superior capacity to predict cancer outcomes relative to conventional clinicopathological features, have been developed [22–29]. While similar signatures have been developed for EEC [30–32], to the best of our knowledge, rare is available for USC.

Here, we carried out a genome-wide search for dysregulated genes in datasets from TCGA (The Cancer Genome Atlas) and GTEx (Genotype-Tissue Expression) and uncovered a 4-gene prognostic signature for USC. As an independent indicator of USC prognosis, this signature performs better than conventional prognostic factors.

## Methods

### Processing of TCGA-USC, GTEx datasets

Level 3 USC RNA-Seq dataset (reads FPKM with HTSeq) along with associated clinical information was downloaded from the TCGA database. Normal uterus GTEx data were downloaded from the UCSC Xena project (http://xena.ucsc.edu/) in October 2019. On the TCGA dataset, cases with follow-up data or overall survival (OS) of less than 30 days were excluded from the study.

#### Identification of dysregulated genes and functional enrichment analysis

Limma, an “R” Bioconductor package was used to identify genes that are dysregulated in USC tissues relative to normal uterine tissue by applying a threshold of |log_2_FC| > 2 and FDR < 0.01. GO (gene ontology) term analysis and KEGG (Kyoto Encyclopedia of Genes and Genomes pathway enrichment analysis were conducted using the ClusterProfiler package on “R”. A *P value* = < 0.05 was considered indicative of significantly enriched functional annotations.

#### Construction and evaluation of the prognostic model

Half of the USC cases were randomly assigned to the training set. Cases with complete records on clinicopathological features, including OS, age, invasion, node, and stage, were assigned to the testing set. In the training set, dysregulated genes with prognostic potential were identified by univariable Cox regression analysis using the Survival package in “R”. *P-value* = < 0.05 was considered significant. To identify the most important prognostic genes, the least absolute shrinkage and selection operator (LASSO) regression method was executed in “R” using the Glmnet package. The prognostic signature for predicting OS was developed through multivariable Cox regression analysis using the “R” Survival package. The prognostic signature was applied in the calculation of the patients’ risk scores. The cases were then ranked into the high-risk and low-risk groups based on the median score. Kaplan-Meier survival analysis was done using the “R” Survival package to plot the survival curves for the 2 risk groups. Receiver operating characteristic (ROC) curve analysis done using the “R” Survival ROC package to test the 4-gene signature’s accuracy in predicting OS for the high and low-risk USC cases. To validate the effectiveness of the signature, the OS risk score for each patient in the testing set was calculated using the signature, followed by Kaplan-Meier curve analysis and ROC estimation as was done in the training set. To evaluate the superiority of the 4-gene signature as a prognostic indicator, ROC curve analysis was done on other clinicopathological features, including age at diagnosis, myometrium invasion, node metastasis and stage. The process outlined above was used to test the signature’s effectiveness at predicting recurrence-free survival (RFS).

## Results

### TCGA-USC patient characteristics

A dataset of 110 UCS samples and 35 adjacent normal uterus tissue samples was downloaded from TCGA. The training and testing set consisted of data from 56 and 74 USC cases, respectively. The clinicopathological features among the 2 groups and the whole dataset did not differ significantly (*P-value* = > 0.05). These features were summarized in Table 1.

**Table 1:** Clinicopathological characteristics of USC patients in this study

Parameters		TCGA set	Training set	Testing set	*P*-value
		*n* = 110	*n* = 56	*n* = 74	
Age, years(mean ± SD)		68.6 ± 1.6	66.8 ± 2.0	67.8 ± 2.0	0.395^b^
Myometrium invasion, percentage (mean ± SD)		50.1 ± 7.3	50.2 ± 10.1	49.3 ± 8.2	0.985^b^
Stage, no(%)	Early (I + II)	53 (48.2)	26 (46.4)	32 (43.2)	0.805 ^c^
	Late (III + IV)	57 (51.8)	30 (53.6)	42 (56.8)	
Lymph node metastasis, no(%)	YES	34 (38.6)^a^	18 (39.1) ^a^	31 (41.9) ^a^	0.908 ^c^
	NO	54 (61.4) ^a^	28 (60.9) ^a^	43 (58.1) ^a^	
Vital status, no(%)	Alive	74 (67.3)	40 (71.4)	48 (64.9)	0.729 ^c^
	Dead	36 (32.7)	16 (28.6)	26 (35.1)	

^a^Value relate to cases with lymph nodes evaluated

^b^ANOVA test

^c^Wilcox test

### Identification of dysregulated genes in USC and functional enrichment analysis

**Fig. 1:**
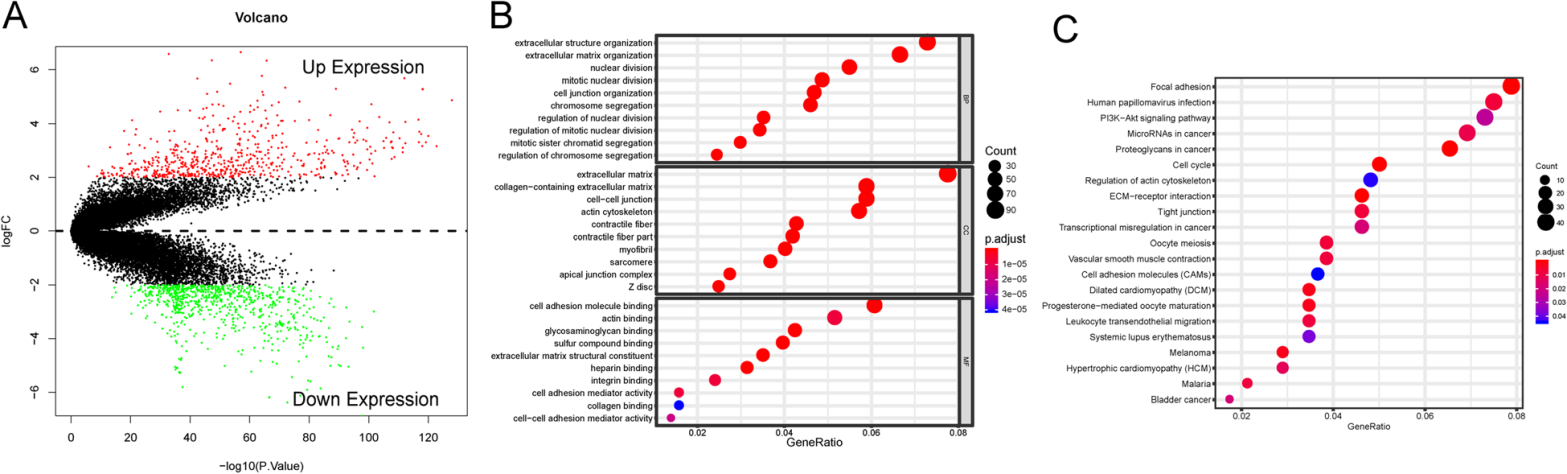
Dysregulated genes in the USC and functional enrichment analysis. **a** Volcano plot shows 1385 up- and down-regulated genes between 110 USCs and 113 normal uterus tissue with the threshold of |log_2_FC| > 2 and FDR < 0.01. **b** Top 30 Gene ontology (GO) biological processes of dysregulated genes. **c** Top 20 Kyoto Encyclopedia of Genes and Genomes (KEGG) pathways of dysregulated genes

To ensure that our analysis compared equivalent numbers of USC and non-USC cases, we downloaded a dataset of normal uterus tissue samples from GTEx (*n* = 78), which along with the 35 in the TCGA dataset brought the total number of normal uterine cases to 113. Using Limma package in “R”, and a cutoff threshold of |log_2_FC| > 2, FDR < 0.01, 1385 genes were identified as being dysregulated in USC tissue vs the normal controls (Fig. 1a). Functional enrichment analysis revealed that the dysregulated genes are significantly associated with 717 GO term processes and 21 KEGG pathways. The most significantly enriched GO terms were extracellular matrix, mitosis, and cell adhesion, processes that might promote cancer progression (Fig. 1b). The most significantly enriched pathways are involved in cell adhesion, cell cycle, PI3K-Akt signaling pathway, cancerous microRNAs, transcriptional misregulation, and pathways involved in melanoma and bladder cancer (Fig. 1c).

### Prognostic signature construction and evaluation in the training set

**Fig. 2:**
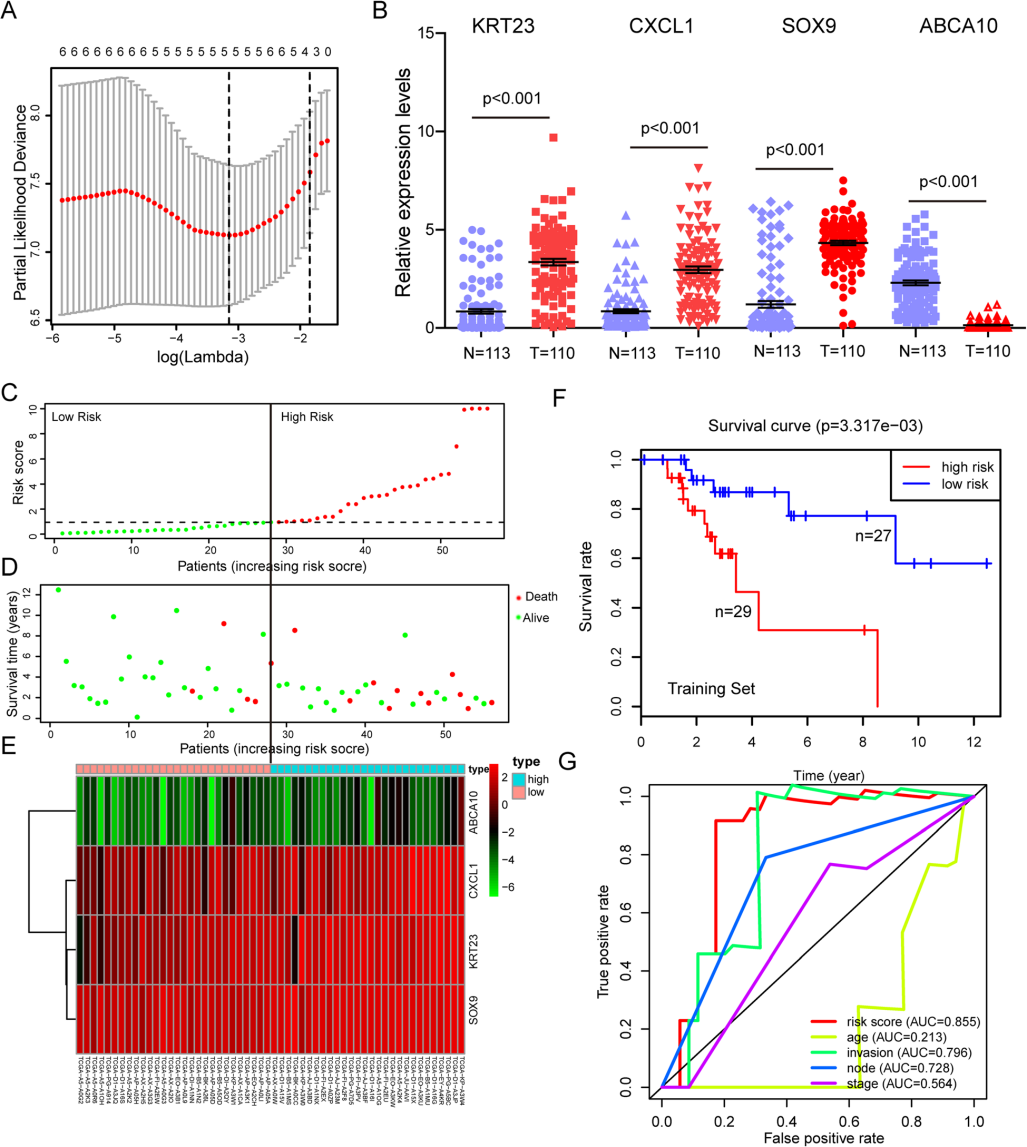
Identification of the 4-gene signature and prediction of overall survival (OS) in the training set. **a** LASSO regression defines 5 critical survival prognostic genes. **b** Expression differences of the 4 genes, identified by multivariable Cox regression analysis, between 110 USC patients and 113 normal endometrium tissues are analyzed by Wilcox test. *P* < 0.05 is considered statistically significant. **c**-**e** The distribution of risk score, OS, and survival status and the 4 genes expression patterns for the 56 patients in the training set. **f** Kaplan–Meier analysis to compare OS between patients in the high- and low-risk group in the training set. **g** ROC analysis of the 4-gene signature and other clinicopathological parameters (age, invasion, node metastasis and stage) for prediction OS in the training set

To identify dysregulated genes that may be associated with OS, we performed univariable Cox regression analysis and uncovered 29 genes that significantly correlated with OS (Table S1). To narrow down to the most important prognostic genes, we used LASSO regression analysis, which revealed 5 dysregulated genes as being potential critical indicators of USC survival (Fig. 2a). Next, multivariable Cox regression analysis narrowed down to a signature 4 genes, KRT23, CXCL1, SOX9 and ABCA10 (Fig. 2b) that effectively predict OS (Table 2). Among these, KRT23, CXCL1, and SOX9 exhibited positive regression coefficients, indicating a high risk of mortality. While ABCA10 showed a negative regression coefficient, implying a low mortality risk. Next, we constructed the following risk prediction formula based on the 4 prognostic genes and used it to calculate each patient’s risk score in the training set: risk score = (0.5424 × expression level of KRT23) + (0.2398 × expression level of CXCL1) + (0.5398 × expression level of SOX9) - (1.7023 × expression level of ABCA10). This signature was used to calculate risk scores for 56 USC cases (individually) in the training set. The risk scores were then ranked linearly and assigned as high-risk or low-risk based on whether they were higher or lower than the median risk score (Fig. 2c). The relationship between risk scores and survival time was showed in Fig. 2d. Visualization of the expression of the 4 genes in a heatmap revealed that the expression level of the 3 high-risk genes increasing with rising risk scores, while the low-risk gene showed an opposite correlation (Fig. 2e). Kaplan-Meier analysis revealed that patients in high-risk group experienced worse outcomes relative to the low-risk group (*P-value* = 0.003317, Fig. 2f). Relative to standard clinicopathological parameters like age, myometrium invasion, node metastasis and disease stage, the 4-gene prognostic signature scored 0.855 in AUC (area under the ROC curve) analysis, indicating superior performance over conventional prognostic factors (0.213, 0.796, 0.728 and 0.564 for age, myometrium invasion, node metastasis and stage, respectively; Fig. 2g).

**Table 2:** Four signature genes constructed in this model

gene	coef^a^	HR	HR.95 L	HR.95H	*P*-value^b^
ABCA10	−1.7023	5.4868	0.8160	36.8946	0.0008
KRT23	0.5424	1.7201	1.0986	2.6931	0.0040
CXCL1	0.2398	1.2709	0.9217	1.7526	0.0033
SOX9	0.5398	1.7156	0.8041	3.6602	0.0072

^a^Coefficients derived from multivariable Cox regression analysis

^b^Derived from the univariate Cox regression analysis

### Validation of the 4-gene signature in the testing set

**Fig. 3:**
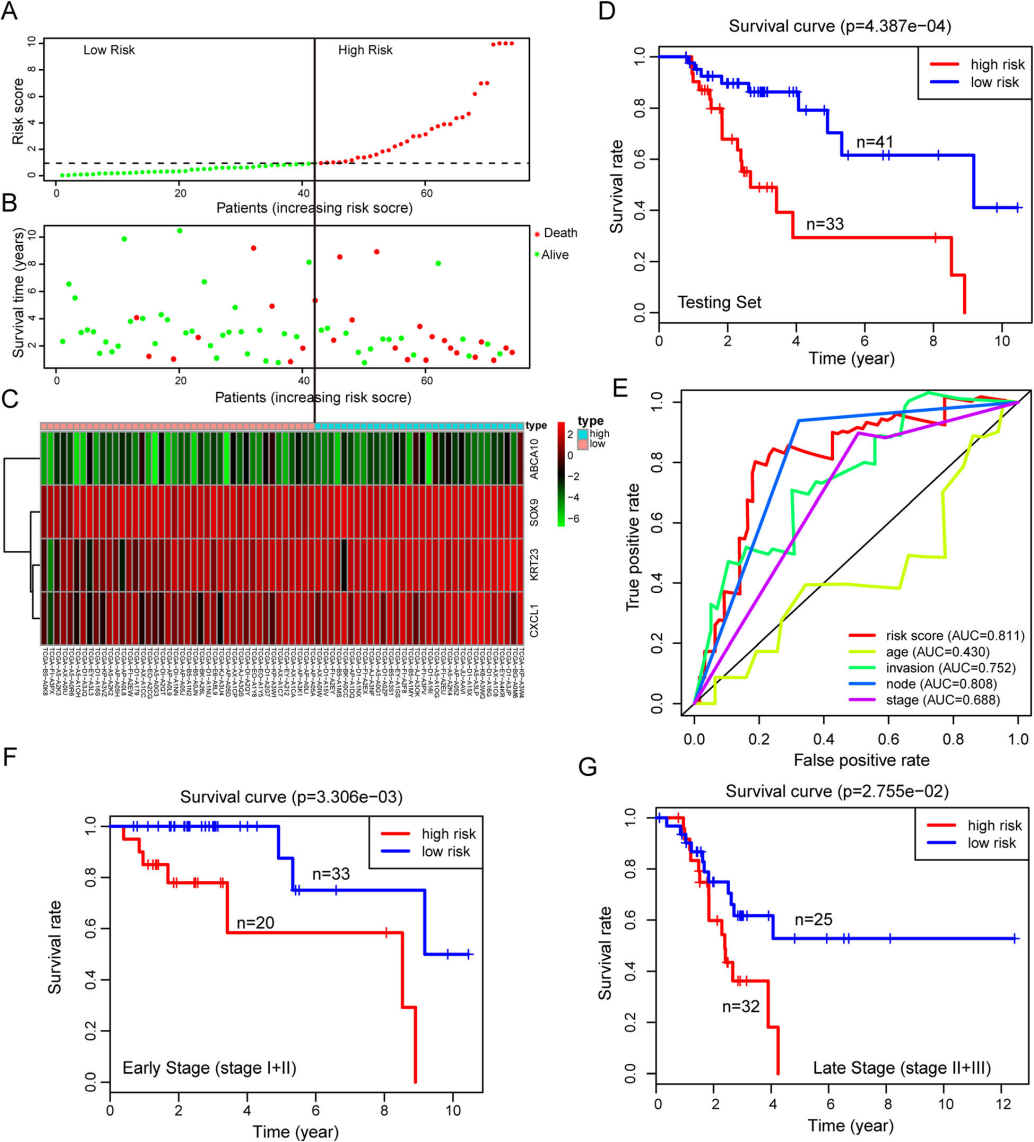
Validation of the 4-gene signature in predicting OS in the testing set and different stage patients. **a**-**c** The distribution of risk score, OS, and survival status and the 4 genes expression patterns for the 74 patients in the training set. **d** Kaplan–Meier analysis compares OS between patients in the high- and low-risk group in the testing set. **g** ROC analysis of the 4-gene signature and other clinicopathological parameters (age, invasion, node metastasis and stage) for prediction OS in the testing set. **f** Kaplan-Meier analysis compares OS between patients in the high- and low-risk group in early stage (I + II) patients. **g** Kaplan-Meier analysis compares OS between patients in the high- and low-risk group in late stage (III + IV) patients

To assess the robustness of the 4-gene prognostic signature, risk scores for the 74 USC cases in the testing set were calculated and ranked as described in section 3.3 (Fig. 3a). The relationship between risk scores and survival is shown in Fig. 3b. This analysis revealed that the expression of the 3 high-risk genes increased with rising risk scores, while the low-risk gene exhibited the opposite effect (Fig. 3c). Kaplan-Meier curve indicated that the high-risk group experienced worse outcomes relative to the low-risk group (*P-value* = 0.0004387, Fig. 3d). The score of 0.811 for the 4-gene signature was revealed by AUC analysis was higher than for conventional prognosis indicators (0.430, 0.752, 0.808 and 0.688 for age, myometrium invasion, node metastasis and stage, respectively; Fig. 3e), consistent with observations made in the training set.

### Independent prognostic value of the 4-gene signature

To evaluate the potential of the 4-gene signature independently of conventional prognosis indicators, we used univariate and multivariate Cox regression analysis on testing set cases with reporting complete clinical features. This analysis revealed that our prognostic signature and tumor stage are both independent predictors of OS (Table 3). Next, we tested if the 4-gene signature could predict OS at different disease stages. To this end, we stratified the cases by stage into early (stage I + II) and late stage (stage III + IV). Patients in high-risk group in both early and late stage exhibited lower OS relative to those in the low-risk group (*P value =* 0.003306 and *P value =* 0.02755, respectively, Fig. 3f-g). These results indicate that the 4-gene signature has superior performance in early stage, highlighting its potential clinical application.

**Table 3:** Univariate and multivariate Cox regression analysis of OS in USC patients in the testing set (*n* = 74)

Parameters	Univariate analysis				Multivariate analysis			
	HR	HR.95 L	HR.95H	*P*-value	HR	HR.95 L	HR.95H	*P*-value
invasion (>50%/≤50%)	1.0203	1.0069	1.0340	0.0029	1.0080	0.9931	1.0232	0.2953
node (YES/NO)	3.6689	1.5769	8.5366	0.0026	2.0157	0.6958	5.8396	0.1965
stage (III + IV/I + II)	2.0795	1.3531	3.1959	0.0008	1.8088	1.0063	3.2510	0.0476
4-gene riskScore (high/low)	1.0511	1.0154	1.0881	0.0047	1.0511	1.0108	1.0930	0.0125

### Evaluation of the 4-gene signature in predicting RFS

**Fig. 4:**
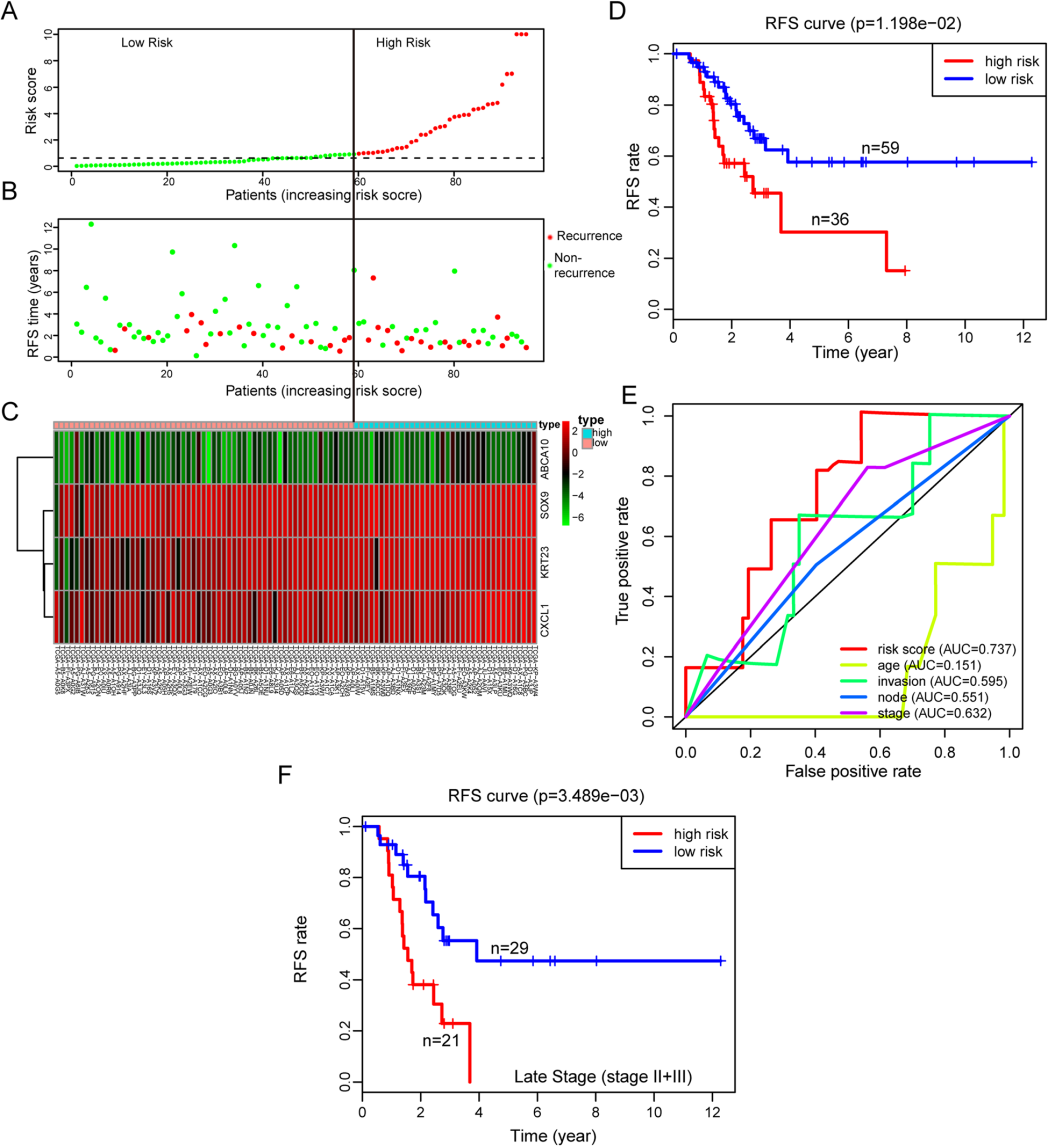
Validation of the 4-gene signature in predicting recurrence-free survival (RFS) in the TCGA dataset. **a**-**c** The distribution of risk score, RFS, and survival status and the 4 genes expression patterns for the 95 patients with RFS data. **d** Kaplan–Meier analysis compares RFS between patients in the high- and low-risk group. **e** ROC analysis of the 4-gene signature and other clinicopathological parameters (age, invasion, node metastasis and stage) for prediction RFS. **f** Kaplan-Meier analysis compares RFS between patients in the high- and low-risk group in late stage (III + IV) patients

To evaluate whether the 4-gene signature could predict recurrence-free survival (RFS) in USC, TCGA-USC cases with RFS data were analyzed. Cases with RFS of < 30 days were excluded and 95 cases further analyzed. Each patient’s risk scores were calculated and ranked as described in section 3.3 (Fig. 4a). The risk scores and recurrent time are shown in Fig. 4b. This analysis revealed that expression of the 4 genes increased with rising risk scores (Fig. 4c). Kaplan-Meier analysis revealed that the high-risk group had higher recurrence rate relative to the low-risk group (*P value* = 0.01198, Fig. 4d). The AUC analysis of the prognostic signature revealed a score of 0.737 at RFS prediction, which was higher than the scores from conventional indicators (0.151, 0.595, 0.551 and 0.632 for age, myometrium invasion, node metastasis and stage, respectively, Fig. 4e). Univariate and multivariate Cox regression analysis revealed the prognostic signature and stage as independent prognostic factors for RFS, consistent with OS analysis (Table 4). Analysis of the effectiveness of the 4-gene signature in predicting RFS at different disease stages revealed that patients in low-risk and high-risk groups had significantly different RFS in late stage (*P value* = 0.003489, Fig. 4f). However, there was no difference in early stage between the two risk groups (Fig. S1).

**Table 4:** Univariate and multivariate Cox regression analysis of RFS in USC patients in USC patients in TCGA cohort (*n* = 95)

Parameters	Univariate analysis				Multivariate analysis			
	HR	HR.95 L	HR.95H	*P*-value	HR	HR.95 L	HR.95H	*P*-value
invasion (>50%/≤50%)	1.0100	0.9981	1.0220	0.1005	1.0010	0.9885	1.0136	0.8808
node (YES/NO)	1.9975	0.9192	4.3409	0.0806	1.2448	0.5153	3.0072	0.6266
stage (III + IV/I + II)	1.9975	1.3016	3.0656	0.0015	1.9088	1.1905	3.0605	0.0073
4-gene riskScore (high/low)	1.0744	1.0248	1.1264	0.0029	1.0735	1.0212	1.1284	0.0053

## Discussion

Here, we analyzed USC datasets from TCGA and GTEx and uncovered 1385 genes that are dysregulated USC tissues relative to normal endometrial tissue. KEGG pathway analysis revealed that these genes mainly belong to cancer-associated pathways, including melanoma and bladder cancer as well as in pathways associated with cell adhesion, cell cycle, PI3K-Akt signaling pathway, cancer-linked microRNAs and transcriptional misregulation. Disruption of cell adhesion may explain why USC tends to disseminate early, spreading to fallopian tubes or invading lymph-vascular space. The tumor suppressor, TP53 is the frequently mutated gene in USC [33]. USC’s high proliferative rate dysregulated cycle control may contribute to the high relapse and mortality rates in endometrial cancers. The PI3K/AKT/mTOR signaling pathway is the most frequently dysregulated pathway in EEC [33]. In USC, PIK3CA mutation occurs in about 30% of cases [11, 33, 34], which is consistent with the involvement of the PI3K-Akt signaling pathway seen from our analysis. Inhibition of PI3K/AKT/mTOR signaling strongly suppresses EEC progression [35–37] and clinical trials targeting PI3K/AKT/mTOR signaling in solid tumors have shown promise [38]. However, the benefits of this against in endometrial cancers is controversial due to the complexity of pharmacological action and toxicity [39]. Further studies are needed to better target PI3K/AKT/mTOR signaling in endometrial cancer.

The TGCA database, which offers a collection of complete transcriptomic data and associated clinical information, is publicly available for data mining [40]. To identify important dysregulated genes associated with USC outcomes, we used LASSO and Cox regression analysis. LASSO is widely applied in modeling high-dimensional data and avoids overfitting risk and improves prediction accuracy [41]. Our analysis generated a 4-gene signature for predicting USC OS by calculating each patient’s risk score. We find that patients with high scores exhibit poor outcomes relative to those with low scores, an observation that was validated in both the training and testing sets. ROC curve analysis revealed this signature’s superiority over conventional prognostic parameters (age, myometrium invasion, node metastasis, and stage) in the training and testing sets. Our data show that both the 4-gene signature and disease stage are independent prognostic indicators OS. Patients with late-stage of the disease have an unfavorable prognosis for most malignant solid tumors. However, for USC, the early-stage disease does not necessarily correlate with good prognosis due to the tumor’s propensity for shedding, spreading and invading the lymph-vascular space even when the lesion confined to the endometrium or polyps. Management of patients with early stage USC is controversial [4, 42, 43]. Our signature identified high-risk patients in the early stage USC group who had much poorer OS relative to low-risk patients in the same group. Our data show that this signature performed better in the early stage group than in the late stage group, highlighting its potential value in guiding the management for early stage USC.

The average recurrence rate for stage IA USC after chemotherapy, radiotherapy or surgery is 8.7, 25 and 12.4% respectively. For stage IB/IC the corresponding recurrence rate are 10.8, 36.6 and 37.3%, respectively [11]. Our 4-gene signature predicts a higher recurrence risk in the high-risk group relative to the low-risk group. Consistently with our OS, ROC curve analysis, this 4-gene signature exhibited superior effectiveness over conventional indicators of RFS. Both the signature and disease stage were an independent prognostic factor for RFS. Our data show that the 4-gene signature is effective at RFS prediction in late stage disease but showed no difference between high and low-risk groups in early stage. This may be due to too few recurrent cases (8 cases out of 45 cases) in early stage in the TCGA cohort.

The 4 genes in the signature have been associated with various cancers. KRT23 has been implicated as an oncogene in liver cancer [44] and colorectal cancer [45]. CXCL1 is overexpressed in EEC tissue relative to normal endometrium and promotes tumorigenesis by promoting neutrophil chemotaxis [46]. Snail induces ovarian epithelial-mesenchymal transition via CXCL1 and CXCL2, representing an immunological therapeutic target [47]. SOX9 overexpression in uterine epithelium may induce endometrial hyperplastic lesions [48], promoting endometrial cancer cell proliferation [49]. ABCA10 has been proposed as a prognostic marker in ovarian carcinoma [50]. Germline single nucleotide polymorphisms in ABCA10 may affect follicular lymphoma overall survival [51]. So far, none of the 4 genes has been associated with USC, though CXCL1 and SOX9 are associated with EEC progression.

## Conclusion

Here, we an analysis of USC genome-wide expression profiles in TCGA and GTEx datasets. We have identified genes that are dysregulated in USC and explored their molecular functions and pathways. More importantly, we have developed and validated a 4-gene signature that robustly predicts USC OS and RFS. This signature is an independent prognostic indicator that is more superior to conventional indicators of USC prognosis, especially when predicting OS in early stage of USC. Our findings highlight the potential of this signature as a guide for personalized USC treatment. However, more independent cohorts are needed to validate the signature and to elucidate the molecular mechanisms of these predictive genes in USC.

## Supplementary Information

**Additional file 1.**

## Data Availability

All datasets generated for this study are included in the manuscript.

## Abbreviations

USC: Uterine serous carcinoma
TCGA: The cancer genome atlas
GTEx: Genotype-tissue expression
OS: Overall patient survival
RFS: Recurrence-free survival
EEC: Endometrioid carcinoma
GO: Gene ontology
KEGG: Kyoto encyclopedia of genes and genomes
LASSO: The least absolute shrinkage and selection operator
ROC: Receiver operating characteristic

## References

[1] Bray F, Ferlay J, Soerjomataram I (2018). Global cancer statistics 2018: GLOBOCAN estimates of incidence and mortality worldwide for 36 cancers in 185 countries. CA Caner J Clin.

[2] Chen W, Sun K, Zheng R, Zeng H, Zhang S, Xia C, Yang Z, Li H, Zou X, He J (2018). Cancer incidence and mortality in China, 2014. Chin J Cancer Res.

[3] Hendrickson M, Ross J, Eifel P (1982). Uterine papillary serous carcinoma: a highly malignant form of endometrial adenocarcinoma. Am J Surg Pathol.

[4] Fader AN, Boruta D, Olawaiye AB, Gehrig PA (2010). Uterine papillary serous carcinoma: epidemiology, pathogenesis and management. Curr Opin Obstet Gynecol.

[5] Hu S, Hinson JL, Matnani R, Cibull ML, Karabakhtsian RG (2018). Are the uterine serous carcinoma underdiagnosed? Histomorphologic and immunohistochemical correlates and clinical follow up in high-grade endometrial carcinomas initially diagnosed as high-grade endometrioid carcinoma. Mod Pathol.

[6] Murali R, Davidson B, Fadare O (2019). High-grade endometrial carcinomas: morphologic and immunohistochemical features, diagnostic challenges and recommendations. Int J Gynecol Pathol.

[7] Yemelyanova A, Ji H (2009). Shih IeM, et al. utility of p16 expression for distinction of uterine serous carcinomas from endometrial endometrioid and endocervical adenocarcinomas: immunohistochemical analysis of 201 cases. Am J Surg Pathol.

[8] Black JD, English DP, Roque DM, Santin AD (2014). Targeted therapy in uterine serous carcinoma: an aggressive variant of endometrial cancer. Womens Health (Lond).

[9] del Carmen MG, Birrer M, Schorge JO (2012). Uterine papillary serous cancer: a review of the literature. Gynecol Oncol.

[10] Holman LL, Pal N, Iglesias DA, etc. Factors prognostic of survival in advanced-stage uterine serous carcinoma Gynecol Oncol (2017) 146:27–33. 10.1016/j.ygyno.2017.04.018 PMC5539404 28465008

[11] Fader AN, Santin AD, Gehrig PA (2013). Early stage uterine serous carcinoma: management updates and genomic advances. Gynecol Oncol.

[12] Slomovitz BM, Burke TW, Eifel PJ (2003). Uterine papillary serous carcinoma (UPSC): a single institution review of 129 cases. Gynecol Oncol.

[13] Hanley KZ, Fadare O, Fisher KE (2016). Clinical significant of positive pelvic washings in uterine papillary serous carcinoma confined to an endometrial polyp. Int J Gynecol Pathol.

[14] Semaan A, Mert I, Munkarah AR (2013). Clinical and pathologic characteristics of serous carcinoma confined to the endometrium: a multi- institutional study. Int J Gynecol Pathol.

[15] Hui P, Kelly M, O’Malley DM (2005). Minimal uterine serous carcinoma: a clinicopathological study of 40 cases. Mod Pathol.

[16] Fader AN, Starks D, Gehrig PA (2009). An updated clinicopathologic study of early-stage uterine papillary serous carcinoma. Gynecol Oncol.

[17] Kommoss F, Faruqi A, Gilks CB (2017). Uterine serous carcinomas frequently metastasize to the fallopian tube and can mimic serous tubal intraepithelial carcinoma. Am J Surg Pathol.

[18] Snyder MJ, Bentley R, Robboy SJ (2006). Transtubal spread of serous adenocarcinoma of the endometrium: an underrecognized mechanism of metastasis. Int J Gynecol Pathol.

[19] Solmaz U, Ekin A, Mat E (2016). Analysis of clinical and pathological characteristics, treatment methods, survival, and prognosis of uterine papillary serous carcinoma. Tumori..

[20] Singh N, Hirschowitz L, Zaino R (2019). Pathologic prognostic factors in endometrial carcinoma (other than tumor type and grade). Int J Gynecol Pathol.

[21] Bradner JE, Hnisz D, Young RA (2017). Transcriptional addiction in Cancer. Cell..

[22] Ozawa T, Kandimalla R, Gao F (2018). A microRNA signature associated with metastasis of T1 colorectal cancers to lymph nodes. Gastroenterology.

[23] Beck D, Thoms JAI, Palu C (2018). A 4-gene lincRNA expression signature predicts risk in multiple cohorts of acute myeloid leukemia patients. Leukemia.

[24] Bagnoli M, Canevari S, Califano D (2016). Development and validation of a microRNA-based signature (MiROvaR) to predict early relapse or progression of epithelial ovarian cancer: a cohort study. Lancet Oncol.

[25] Rini B, Goddard A, Knezevic D (2015). A 16-gene assay to predict recurrence after surgery in localized renal cell carcinoma: development and validation studies. Lancet Oncol.

[26] Li J, Chen Z, Tian L (2014). LncRNA profile study reveals a three-lncRNA signature associated with the survival of patients with esophageal squamous cell carcinoma. Gut.

[27] Verhaak RG, Tamayo P, Yang JY (2013). Prognostically relevant gene signatures of high-grade serous ovarian carcinoma. J Clin Invest.

[28] Sandoval J, Mendez-Gonzalez J, Nadal E (2013). A prognostic DNA methylation signature for stage I non-small-cell lung cancer. J Clin Oncol.

[29] Li X, Zhang Y, Zhang Y (2010). Survival prediction of gastric cancer by a seven-microRNA signature. Gut.

[30] Zhou M, Zhang Z, Zhao H (2018). A novel lncRNA-focus expression signature for survival prediction in endometrial carcinoma. BMC Cancer.

[31] Farkas SA, Sorbe BG, Nilsson TK (2017). Epigenetic changes as prognostic predictors in endometrial carcinomas. Epigenetics..

[32] Torres A, Torres K, Pesci A (2013). Diagnostic and prognostic significance of miRNA signatures in tissues and plasma of endometrioid endometrial carcinoma patients. In J Cancer.

[33] Jones NL, Xiu J, Chatterjee-Paer S (2017). Distinct molecular landscapes between endometrioid and nonendometrioid utrine carcinomas. Int J Cancer.

[34] Catasus L, D'Angelo E, Pons C (2010). Expression profiling of 22 genes involved in the PI3K-AKT pathway identifies two subgroups of high-grade endometrial carcinomas with different molecular alterations. Mod Pathol.

[35] Packer LM, Geng X, Bonazzi VF (2017). PI3K inhibitors synergize with FGFR inhibitors to enhancer antitumor responses in FGFR2 mutant endometrial cancers. Mol Cancer Ther.

[36] Suh DS, Park SE, Jin H (2018). LRIG2 is a growth suppressor of Hec-1A and Ishikawa endometrial adenocarcinoma cells by regulating PI3K/AKT- and EGFR-mediated apoptosis and cell-cycle. Oncogenesis..

[37] Hsu AH, Lun MA, Shim KS (2018). Crosstalk between PKCα and PI3K/AKT signaling is tumor suppressive in the endometrium. Cell Rep.

[38] Bendell JC, Varghese AM, Hyman DM (2018). A first-in- human phase 1 study of LY3023414, an oral PI3K/mTOR dual inhibitor, in patients with advanced cancer. Clin Cancer Res.

[39] Barra F, Evangelisti G, Ferro Desideri L (2019). Investigational PI3K/AKT/mTOR inhibitors in development for endometrial cancer. Expert Opin Investig Drugs.

[40] Ali MM, Akhade VS, Kosalai ST, etc. PAN-cancer analysis of S-phase enriched lncRNAs identifies oncogenic drivers and biomarkers Nat Commun (2018) 9:883. 10.1038/s41467-018-03265-1 PMC5830406 29491376

[41] Pi L, Halabi S (2018). Combined performance of screening and variable selection methods in ultra-high dimensional data in predicting time-to-event outcomes. Diagn Progn Res.

[42] Wang Y, Yu M, Yang JX (2018). Clinicopathological and survival analysis of uterine papillary serous carcinoma: a single institutional review of 106 cases. Cancer Manag Res.

[43] Black C, Feng A, Bittinger S (2016). Uterine papillary serous carcinoma: a single-institution review of 62 cases. Int J Gynecol Cancer.

[44] Kim D, Brocker CN, Takahashi S (2019). Keratin 23 is a peroxisome proliferator-activated receptor alpha-dependent, MYC-amplified oncogene that promotes hepatocyte proliferation. Hepatology.

[45] Zhang N, Zhang R, Zou K (2017). Keratin 23 promotes telomerase reverse transcriptase expression and human colorectal cancer growth. Cell Death Dis.

[46] Wallace AE, Sales KJ, Catalano RD (2009). Prostaglandin F2alpha-F-prostanoid receptor signaling promotes neutrophil chemotaxis viachemokine (C-X-C motif) ligand 1 in endometrial adenocarcinoma. Cancer Res.

[47] Taki M, Abiko K, Baba T (2018). Snail promotes ovarian cancer progression by recruiting myeloid-derived suppressor cell via CXCR2 ligand upregulation. Nat Commun.

[48] Gonalez G, Mehra S, Wang Y (2016). Sox9 overexpression in uterine epithelia induces endometrial gland hyperplasia. Differentiation.

[49] Saegusa M, Hashimura M, Suzuki E (2012). Transcriptional up-regulation of Sox9 by NF-κB in endometrial carcinoma cells, modulating cell proliferation through alteration in the p14(ARF)/p53/p21(WAF1) pathway. Am J Pathol.

[50] Wang Y, Lei L, Chi YG (2019). A comprehensive understanding of ovarian carcinoma survival prognosis by novel biomarkers. Eur Rev Med Pharmacol Sci.

[51] Baecklund F, Foo JN, Bracci P (2014). A comprehensive evaluation of the role of genetic variation in follicular lymphoma survival. BMC Med Genet.

