# Supplementary material for: A 4-gene signature predicts prognosis of uterine serous carcinoma
Source: BMC Cancer. 2021 Feb 12;21:154. doi: 10.1186/s12885-021-07834-4 (PMC7881619; doi:10.1186/s12885-021-07834-4)
Supplement: Supplementary file 1 — Additional file 1. [file 12885_2021_7834_MOESM1_ESM.docx]

Table S1: Univariate Cox regression analyses of the 29 dysregulated genes associated with overall survival in USC

| gene | HR | HR.95L | HR.95H | *P*-value |
| --- | --- | --- | --- | --- |
| ABCA9 | 12.62384 | 3.124287 | 51.00728 | 0.000372 |
| ABCA10 | 21.85341 | 3.591192 | 132.9841 | 0.000815 |
| CXCL1 | 1.497172 | 1.143599 | 1.96006 | 0.003323 |
| KRT23 | 1.831878 | 1.213274 | 2.765886 | 0.003982 |
| TRPC4 | 13.2422 | 2.159895 | 81.18722 | 0.005234 |
| SOX9 | 2.051129 | 1.214391 | 3.464394 | 0.007225 |
| MMP11 | 1.594378 | 1.103044 | 2.304567 | 0.013075 |
| FOXQ1 | 1.869234 | 1.136763 | 3.073669 | 0.013697 |
| AC126755.1 | 12.87981 | 1.615133 | 102.7095 | 0.015842 |
| ZNF300P1 | 56.6399 | 1.813441 | 1769.056 | 0.021508 |
| MMP10 | 1.303212 | 1.039607 | 1.633659 | 0.021628 |
| RPL10 | 2.707372 | 1.150461 | 6.37124 | 0.022551 |
| SOCS2-AS1 | 5.398615 | 1.241338 | 23.47873 | 0.024562 |
| SVIL | 2.81535 | 1.140036 | 6.952579 | 0.024825 |
| AC159540.2 | 2.20E+11 | 20.54287 | 2.35E+21 | 0.026658 |
| ABCA6 | 307.4621 | 1.866142 | 50656.89 | 0.027842 |
| LYVE1 | 6.290102 | 1.208288 | 32.74501 | 0.028908 |
| CHMP4C | 2.34107 | 1.090145 | 5.027411 | 0.029161 |
| CHRDL1 | 1.62604 | 1.033296 | 2.558808 | 0.035592 |
| MMP7 | 1.261402 | 1.013806 | 1.569467 | 0.037256 |
| IDH2 | 2.240647 | 1.0349 | 4.851191 | 0.040657 |
| SLC22A3 | 1.982124 | 1.027804 | 3.822532 | 0.04117 |
| TSPYL2 | 0.483443 | 0.240497 | 0.971807 | 0.041326 |
| LDB3 | 0.016909 | 0.000331 | 0.86449 | 0.042102 |
| RAMP1 | 1.53299 | 1.013219 | 2.319399 | 0.043164 |
| PROM2 | 1.574128 | 1.012998 | 2.446084 | 0.043655 |
| MGP | 1.574307 | 1.007408 | 2.460217 | 0.046331 |
| IL33 | 1.573165 | 1.005121 | 2.46224 | 0.047445 |
| PRAP1 | 0.689318 | 0.476942 | 0.996261 | 0.047715 |


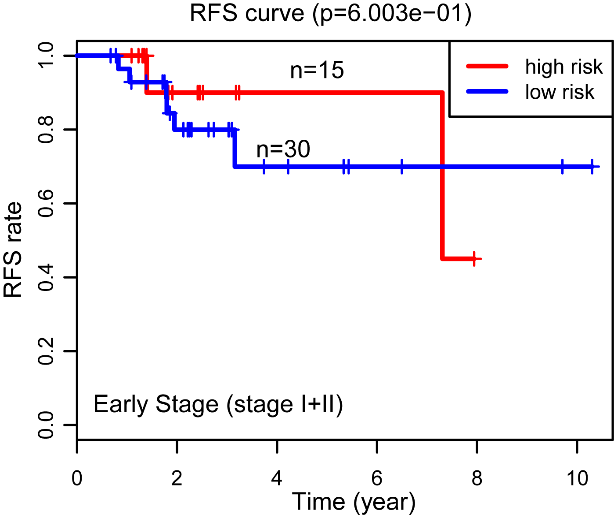


**Figure.S1** Kaplan-Meier analysis compares RFS between patients in the high- and low-risk group in early stage (I+II) patients showing no difference (*P* value＞0.05).
